# Comparative Transcriptome Analysis Reveals the Impact of a High-Fat Diet on Hepatic Metabolic Function in Tilapia (*Oreochromis niloticus*)

**DOI:** 10.3390/ani14223204

**Published:** 2024-11-08

**Authors:** Rui Jia, Yiran Hou, Linjun Zhou, Liqiang Zhang, Bing Li, Jian Zhu

**Affiliations:** 1 Key Laboratory of Integrated Rice-Fish Farming Ecology, Ministry of Agriculture and Rural Affairs, Freshwater Fisheries Research Center, Chinese Academy of Fishery Sciences, Wuxi 214081, China; jiar@ffrc.cn (R.J.); houyr@ffrc.cn (Y.H.); zhoulinjun@ffrc.cn (L.Z.); zhangliqiang@ffrc.cn (L.Z.); 2 Wuxi Fisheries College, Nanjing Agricultural University, Wuxi 214128, China

**Keywords:** metabolic function, transcriptome analysis, high-fat diet, oxidative phosphorylation, *Oreochromis niloticus*

## Abstract

Hepatic steatosis is prevalent among cultured fish, yet the molecular mechanisms remain incompletely understood. This study aimed to assess changes in hepatic metabolic function in tilapia and to explore the underlying molecular mechanisms through transcriptomic analyses. Tilapia were allocated into two groups: a normal control (Ctr)-fed group and a high-fat diet (HFD)-fed group. Serum biochemical analyses revealed that HFD feeding led to liver damage and lipid accumulation, characterized by elevated levels of glutamic-pyruvic transaminase (GPT), glutamic-oxaloacetic transaminase (GOT), triglycerides (TGs), and total cholesterol (TC). Transcriptome analysis showed that 538 genes were significantly downregulated, and 460 genes were significantly upregulated in the HFD-fed fish. Gene Ontology (GO) enrichment analysis showed that these differentially expressed genes (DEGs) were apparently involved in the lipid metabolic process and monocarboxylic acid metabolic process. Meanwhile, Kyoto Encyclopedia of Genes and Genomes (KEGG) enrichment analysis indicated significant alterations in pathways of steroid biosynthesis, porphyrin metabolism, terpenoid backbone biosynthesis, and retinol metabolism after HFD feeding. Additionally, results from Gene Set Enrichment Analysis (GSEA) revealed that gene expression patterns in pathways including oxidative phosphorylation, protein export, protein processing in the endoplasmic reticulum, and ribosome biogenesis were positively enriched in the HFD-fed tilapia. These findings provide novel insights into the mechanisms underlying HFD-induced hepatic dysfunction in fish, contributing to the optimization of feeding strategies in aquaculture.

## Simple Summary

Hepatic steatosis caused by nutritional imbalance is prevalent in cultured fish, leading to metabolic dysfunction, decrease in disease resistance, and increase in mortality rates. Consequently, the molecular mechanisms of hepatic steatosis are critically important to elucidate in fish. This study aimed to assess changes in hepatic metabolic function in tilapia and to explore the underlying molecular mechanisms through transcriptomic analyses. The results indicated that high-fat diet (HFD) feeding significantly altered the lipid metabolic process and monocarboxylic acid metabolic process. Furthermore, HFD feeding caused significant changes in pathways of steroid biosynthesis, porphyrin metabolism, terpenoid backbone biosynthesis, and retinol metabolism. Additionally, results from Gene Set Enrichment Analysis (GSEA) showed that gene expression patterns in pathways including oxidative phosphorylation, protein export, protein processing in the endoplasmic reticulum, and ribosome biogenesis were positively enriched in HFD-fed tilapia. These findings suggest that HFD feeding alters energy metabolism, lipid metabolism, and protein synthesis in the liver of tilapia.

## 1. Introduction

Fat is a crucial nutrient for aquatic animals, supplying energy and essential fatty acids required for growth, development, and health maintenance [1]. In aquaculture practices, temporarily increasing the dietary fat content can effectively enhance the growth rate of farmed species. Furthermore, appropriately increasing the fat level in diets can conserve protein content and reduce production costs [2,3]. Nonetheless, long-term high-fat diets (HFDs) may induce several problems in farmed fish, including slowed growth, immune suppression, inflammation, and excessive liver fat deposition, which in turn can lead to metabolic disorders [4]. The issue of excessive lipid deposition in fish has been a persistent challenge in aquaculture [5]. Fish liver is more susceptible to fat deposition, leading to hepatic steatosis and fatty liver disease, because the liver is the principal organ for lipid metabolism in fish [6,7]. The factors causing hepatic steatosis in farmed fish are complex and multifaceted. The primary cases of hepatic steatosis in cultured fish are attributed to issues related to nutrition of feed [5].

Hepatic steatosis, arising from nutritional imbalances, is widespread among cultured fish worldwide, leading to metabolic dysfunction, reduced disease resistance, and higher mortality rates [8]. Numerous studies have established hepatic steatosis (or liver injury) models via HFD feeding in fish to investigate the disease mechanisms and screen for hepatoprotective drugs [9,10,11]. Specifically, liver damage induced by HFD, characterized by fat accumulation, oxidative stress, inflammatory responses, and necrosis, has been well documented in species like *Danio rerio* and *Oryzias latipes* [12,13]. In *Megalobrama amblycephala*, 8 weeks of HFD feeding heightened liver damage markers, notably glutamate pyruvate transaminase (GPT) and glutamate oxaloacetate transaminase (GOT), while also reducing antioxidant levels [14]. Similarly, HFD feeding reduced growth and disrupted lipid metabolism in the liver of *Cyprinus carpio* [15]. Furthermore, in *Scophthalmus maximus*, HFD feeding led to hepatic oxidative stress and weakened nonspecific immunity, but these effects were mitigated by vitamin E supplementation [16]. HFD feeding also detrimentally affected intestinal function via increasing triglyceride (TG) content and changing microbiota composition [17,18]. Despite increasing research focus on hepatic steatosis (or fatty liver injury) in fish, comprehensive understanding of the underlying mechanisms of the disease remains limited. Therefore, exploring the potential mechanisms of lipotoxicity in fish liver is crucial.

In recent years, transcriptomic approaches have been widely utilized in fish physiology, pathology, and toxicology studies. These methods facilitate comprehensive analyses of gene expression at both structural and functional levels throughout the genome, enhancing our understanding of the molecular regulatory networks that control various biological processes [19]. For example, transcriptome analysis of *Danio rerio* with diet-induced obesity showed that lipid catabolism and fatty acid oxidation predominantly occur in muscle tissues during 5-HT treatment [20]. Furthermore, maternal HFD in *O. latipes* has been shown to alter the egg composition by reducing amino acid catabolism and upregulating the genes associated with endoplasmic reticulum (ER) stress [21]. Similarly, in *Aplodinotus grunniens*, transcriptome analysis showed obvious alterations in pathways associated with immunity, apoptosis, and disease after HFD feeding [22]. These findings underscore the value of transcriptomic analysis in advancing our understanding of pathological processes in fish.

Tilapia (*Oreochromis niloticus*), a tropical freshwater fish, is extensively cultivated globally due to its remarkable adaptability, rapid growth rate, and significant economic benefits. It is particularly valued in China and African countries not only for its high nutritional value but also for its wide adaptability to diverse aquaculture environments and relatively low production costs. In tilapia aquaculture, it is common to observe that the fish are either overfed or given high-fat/high-sugar diets to enhance growth, which may lead to excessive lipid deposition and even fatty liver disease [23]. Our previous research, in conjunction with studies by others, has confirmed that oxidative stress, dysregulated lipid metabolism, and inflammatory responses are involved in HFD-induced liver injury in tilapia [24,25,26,27,28,29]. Nonetheless, the mechanisms through which HFD induces liver injury in tilapia remain incompletely understood. Consequently, employing transcriptomic approaches to explore the fundamental mechanisms behind fatty liver damage in tilapia is considered essential.

## 2. Materials and Methods

### 2.1. Animal and Experimental Design

Tilapia (initial average weight of 40 ± 1.1 g) were sourced from the farm of Freshwater Fisheries Research Center (Wuxi, China). These fish were healthy, uninjured, and free from pathogens. Prior to experimentation, they underwent a two-week acclimation period in a laboratory recirculation aquaculture system. During the acclimation, environmental conditions were carefully controlled. Temperature was maintained between 27 °C and 30 °C, dissolved oxygen level ranged from 5.4 to 6.3 mg/L, and pH level was kept between 7.3 and 7.9. After acclimation, the tilapia were assigned to two dietary groups: a control group (Ctr) fed a normal diet containing 6% fat, and a high-fat diet group (HFD) receiving a diet with 21% fat. Each group, consisting of 30 fish divided equally among three tanks (10 fish per tank), was fed daily with feed amounting to approximately 4% of their body weight, distributed across two feedings. The diets were formulated based on the recipes described in our previous reports [29]. Throughout the study, we observed no fish mortality or abnormal behavior. Moreover, the water quality was consistently maintained within the optimal range for tilapia cultivation (NO_2_^−^ −N < 0.005 mg/L, NH_3_ < 0.02 mg/L).

After a 60-day farming period, nine fish from each of the Ctr and HFD groups were randomly chosen and promptly anesthetized with 100 mg/L MS-222 (AbMole, Shanghai, China). Blood was sampled from each sampled fish to obtain serum through centrifugation (5000× *g*, 10 min, 4 °C). The liver tissue was harvested from each sampled fish and flash-frozen in liquid nitrogen for the transcriptome sequencing. For the transcriptome analysis, liver tissues from three individual fish were pooled to form a single sample, and each group had three replicates [30,31].

### 2.2. Serum Parameter Measurement

The activities of glutamate pyruvate transaminase (GPT) and glutamate oxaloacetate transaminase (GOT) were assessed using the Reitman–Frankel method, facilitated by a spectrophotometer for quantitative analysis. The triglyceride (TG) level was determined using the GPO-PAP method, while total cholesterol (TC) was measured via the COD-PAP method [32].

### 2.3. Transcriptome Sequencing in Liver

Total RNA was extracted from the liver tissues of the Ctr and HFD groups using the Trizol reagent kit (Invitrogen, Carlsbad, CA, USA), following the manufacturer’s instructions. The mRNA was enriched using Oligo (dT)-conjugated magnetic beads and fragmented into short segments using fragmentation buffer. These segments served as templates for synthesizing the first cDNA through reverse transcription. Subsequently, the second cDNA strand was synthesized using a reaction mixture containing buffer, dNTPs, RNase H, and DNA Polymerase I. The resulting double-stranded cDNA was purified with a QiaQuick PCR purification kit (QIAGEN, Valencia, CA, USA). The cDNA underwent end-repair, adenylation, and adapter ligation, followed by construction through PCR amplification. The library was then sequenced on an Illumina HiSeq™2500 system (Gene Denovo Biotechnology Co., Guangzhou, China). The raw sequencing data have been uploaded to the NCBI Open database (PRJNA1170218).

The raw data were filtered to remove reads containing adapters, reads composed entirely of A bases, reads with >10% N bases, and low-quality reads, thereby obtaining high-quality clean reads. Subsequently, ribosomal RNA (rRNA) in the cleaned data was eliminated using Bowtie2 (version 2.2.8). The prossed reads were then aligned to the reference genome of *O. niloticus* (NCBI, PRJNA354796) using Tophat2 (version 2.1.1) [33]. Transcriptome assembly was performed using Cufflinks, enabling the identification of both known and novel transcripts [34]. Following assembly, a detailed analysis and quantification of gene expression were conducted. Differential gene expression analysis between Ctr and HFD groups was performed using the DESeq2 R package (1.20.0) [35], wherein genes were designated as differentially expressed (DEGs) with a threshold: *p* < 0.05 and |log2 fold change (FC)| ≥ 1 [36]. The DEGs were further analyzed for changes in biological functions and key signaling pathways by mapping them to the Gene Ontology (GO) database using Goseq R package (1.46.0) and to the Kyoto Encyclopedia of Genes and Genomes (KEGG) database using KOBAS 2.0 software. Furthermore, gene set enrichment analysis (GSEA) was performed to pinpoint distinct signaling pathways between the Ctr and HFD groups, where thresholds for significance were set at a normalized enrichment score (ES) > 1, a nominal *p*-value < 0.05, and a False Discovery Rate (FDR) < 0.25.

### 2.4. Quantitative Real-Time PCR (qPCR) Assessment

A total of 50 mg of liver tissue from each sample in both the Ctr and HFD groups (3 samples for each group) was used to extract total RNA employing the RNAiso Plus reagent (Code No. 9109; Takara, Beijing, China), supplemented with chloroform and isopropyl alcohol according to the manufacturer’s instructions. The RNA quality was evaluated based on the OD 260/280 absorbance ratio and gel electrophoresis. The extracted RNA (1 μg) was utilized for cDNA synthesis through reverse transcription, employing the PrimeScript™ RT Reagent Kit (Code No. RR047; Takara). The reverse transcription process is conducted in two steps. In the first step, the reaction mixture consists of 1 μg of RNA, 2 μL of 5× gDNA Eraser Buffer, and 1 μL of gDNA Eraser, with RNase Free dH_2_O added to reach a final volume of 10 μL. This mixture is incubated at 42 °C for 2 min to remove genomic DNA. Following the initial incubation, 1 μL of PrimeScript RT Enzyme Mix I, 1 μL of RT Primer Mix, 4 μL of 5× PrimeScript Buffer, and 4 μL of RNase Free dH_2_O are added to the mixture. The reaction is then incubated at 37 °C for 15 min, followed by a brief heating at 85 °C for 5 s to synthesize cDNA.

The expression of the target genes was quantified by qPCR using cDNA as the template on the CFX96 Real-Time PCR Detection System (Bio-Rad, Hercules, CA, USA). The reaction mixture consisted of 12.5 μL of TB Green Premix Ex Taq II (Code No. RR820; Takara), 1 μL each of forward and reverse primers, 2 μL of cDNA, and 8.5 μL of ddH_2_O. The qPCR reaction conditions are as follows: an initial denaturation step at 95 °C for 30 s, followed by 40 cycles of denaturation at 95 °C for 5 s and annealing/extension at 60–62 °C for 30 s. Following these cycles, a melt curve analysis was performed. To ensure reliability, three qPCR replicates have been performed for each biological sample to determine Cq mean values. The Cq value obtained was employed to determine the relative expression of target genes employing the 2^^−ΔΔCq^ method, using *ubce* as the reference gene [37]. The specific primers utilized for this analysis were designed with the Primer-BLAST tool available on NCBI, as detailed in Table 1.

### 2.5. Statistical Analysis

Statistical analysis of all data in the study was conducted using SPSS (v 20.0). Before performing differential analysis, all data underwent normality and variance homogeneity testing using the Shapiro–Wilk test and Levene’s test, respectively. Differences in biochemical parameters and gene expression detected via qPCR between the Ctr and HFD groups were evaluated using Student’s *t*-test, with significance set at *p* < 0.05. Pearson’s method was used to analyze the correlation between qPCR results and transcriptome sequencing results.

## 3. Results

### 3.1. Changes in Serum Biochemical Parameters

After 60 days of feeding, the activities of GPT and GOT were significantly increased in the HFD group compared to the Ctr group (Table 2). Similarly, the levels of TG and TC were also markedly increased after HFD feeding relative to the fish in the Ctr group (Table 2).

### 3.2. Transcriptome Sequencing and Gene Expression

Following filtering, RNA-seq data analysis yielded 32,146,546–50,572,504 high-quality clean reads (Table 3). Quality assessment revealed Q_20_ values ranging from 98.01 to 98.30 and Q_30_ values varied from 92.02% to 95.58%. The GC content varied from 50.01% to 50.69% (Table 3). The overall mapping ratio was between 87.50% and 89.45% (Table 3). In total, 29,550 genes were identified, comprising 24,116 known genes and 354 newly discovered genes.

**Figure 1:**
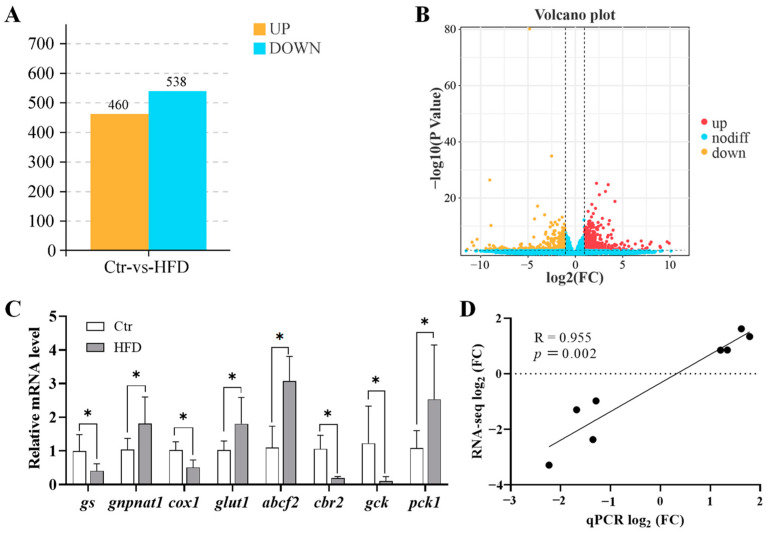
DEGs in tilapia liver: Ctr vs. HFD. (**A**) Number of significantly upregulated and downregulated genes in the liver. (**B**) Volcano plot showing DEGs identified by RNA-seq. (**C**) Validation of RNA-seq data by qPCR analysis, presented as means ± 95% CI (*n* = 3); the relative expression is linear scale. The asterisk “*” denotes significant differences between the Ctr and HFD groups. (**D**) Correlation analysis between qPCR and RNA-seq data.

The examination of DEGs between the Ctr and HFD groups identified a total of 998 DEGs, with 460 increased and 538 decreased in the HFD group relative to the Ctr group (Figure 1A,B). To validate the transcriptome results, qPCR analysis was conducted on eight metabolism-related genes, with findings presented in Figure 1C. This validation demonstrated a substantial correlation between the RNA-seq data and the qPCR results (r = 0.955, *p* = 0.002; Figure 1D), thus indicating the accuracy and reliability of the transcriptome sequencing findings.

### 3.3. GO Enrichment Analysis of DEGs

**Figure 2:**
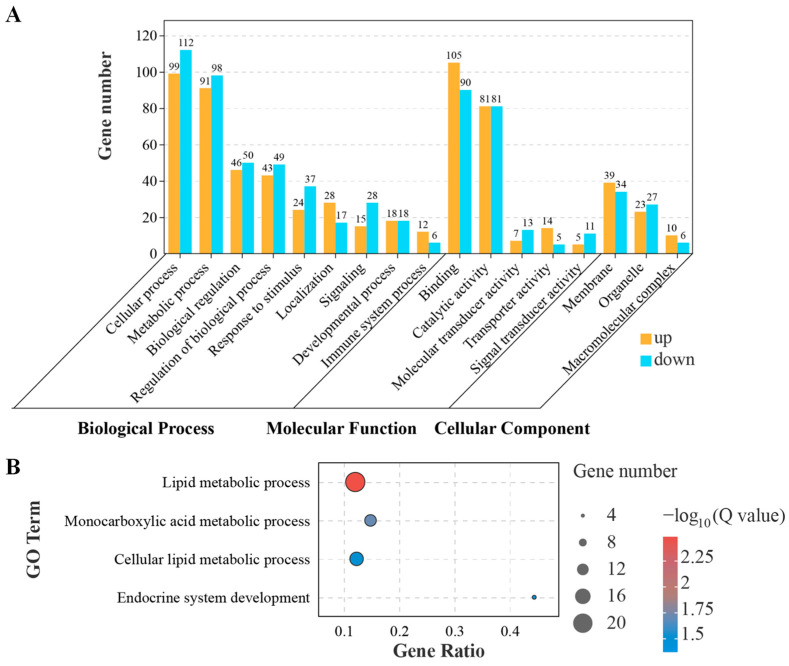
GO enrichment analysis for DEGs in liver after HFD feeding. (**A**) GO enrichment for DEGs in biological process, molecular function and cellular component. (**B**) Significant enriched GO terms for DEGs in biological process category.

The DEGs were enriched in the GO database to evaluate their biological functions. In the biological process category, the DEGs were predominantly enriched in cellular process, metabolic process, and biological regulation (Figure 2A). In the molecular function category, enrichment of DEGs was observed in binding, catalytic activity and molecular transducer activity (Figure 2A). In the cellular component category, DEGs were chiefly enriched in membrane, organelle, and macromolecular complex (Figure 2A). Specifically, the DEGs were significantly enriched in several processes (Figure 2B): lipid metabolic process (*p*.adj = 0.003215), monocarboxylic acid metabolic process (*p*.adj = 0.019524), cellular lipid metabolic process (*p*.adj = 0.030446), and endocrine system development (*p*.adj = 0.041636).

### 3.4. KEGG Enrichment Analysis of DEGs

**Figure 3:**
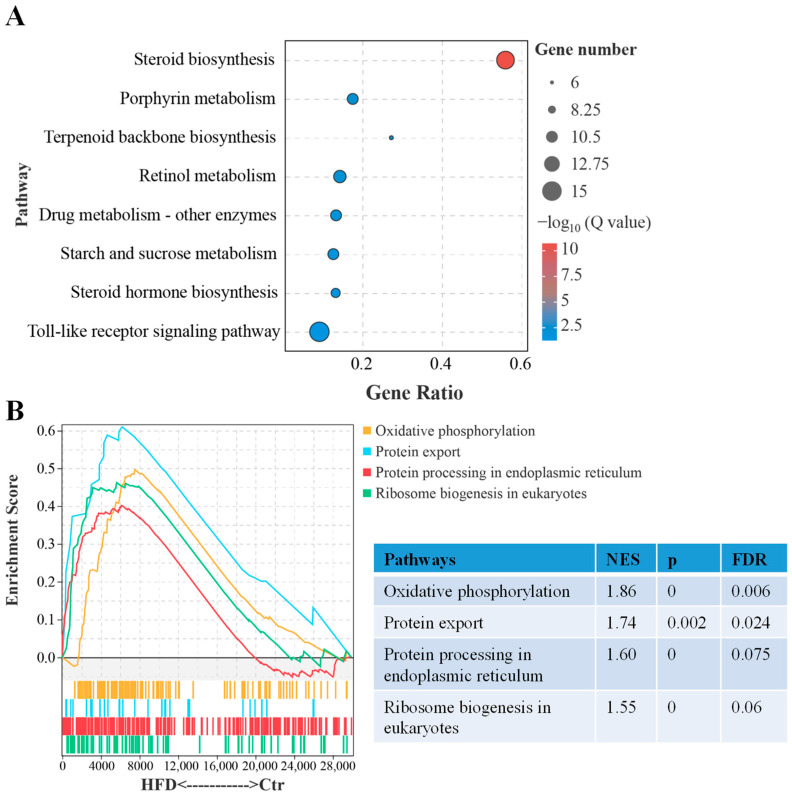
Key signaling pathways in liver of tilapia after HFD feeding. (**A**) KEGG enrichment analysis for the DEGs. (**B**) GSEA of gene expression patterns based on KEGG database.

The KEGG enrichment analysis revealed that 316 DEGs were associated with 133 pathways. Figure 3A lists the top eight enriched pathways (*p*.adj < 0.05), with seven of these pathways related to metabolic functions. Specifically, the pathways include steroid biosynthesis, porphyrin metabolism, terpenoid backbone biosynthesis, retinol metabolism, starch and sucrose metabolism, and steroid hormone biosynthesis. Meanwhile, the GSEA indicated that the gene expression patterns in oxidative phosphorylation, protein export, protein processing in the endoplasmic reticulum, and ribosome biogenesis exhibited an upregulated trend in the HFD group compared to the Ctr group (Figure 3B).

### 3.5. Changes in the Steroid Biosynthesis Pathway

**Figure 4:**
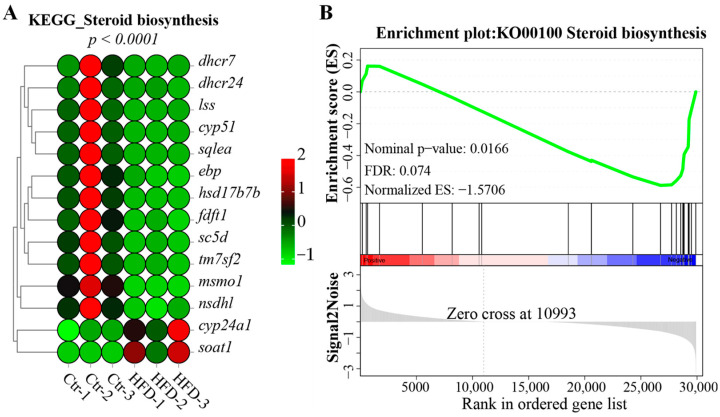
Changes in the steroid biosynthesis pathway in the liver of tilapia after HFD feeding. (**A**) Expression of DEGs in the steroid biosynthesis pathway; *dhcr24*, 24-dehydrocholesterol reductase; *dhcr7*, 7-dehydrocholesterol reductase; *lss*, lanosterol synthase; *cyp51*, lanosterol 14-alpha demethylase; *sqlea*, squalene epoxidase a; *ebp*, EBP cholestenol delta-isomerase; *hsd17b7b*, 3-keto-steroid reductase; *fdft1*, farnesyl-diphosphate farnesyltransferase 1; *sc5d*, sterol-C5-desaturase; *tm7sf2*, transmembrane 7 superfamily member 2; *msmo1*, methylsterol monooxygenase 1; *nsdhl*, NAD(P) dependent steroid dehydrogenase-like, *cyp24a1*, 1,25-dihydroxyvitamin D(3) 24-hydroxylase, mitochondrial; *soat1*, sterol O-acyltransferase 1. (**B**) GSEA of gene expression patterns for the steroid biosynthesis pathway.

After 60 days of feeding, the steroid biosynthesis pathway was significantly changed (*p*.adj < 0.001; Figure 4A). Within this pathway, twelve genes were significantly downregulated and two were clearly upregulated in the fish fed with HFD relative to the Ctr group. Furthermore, GSEA revealed that this pathway was negatively enriched in the HFD-fed fish relative to those on a control diet (Figure 4B).

## 4. Discussion

### 4.1. The Influence of HFD Blood Paramaters

The liver, the principal organ responsible for metabolizing materials and energy in aquatic animals, plays a crucial role in maintaining the overall health of aquatic animals. Dysfunction or damage to the liver in farmed fish not only triggers severe metabolic disorders but has also become a prevalent issue in aquaculture. Notably, HFD is increasingly implicated in contributing to these metabolic irregularities and liver injuries [4]. In this study, tilapia was fed a HFD (21%) for 60 days, resulting in a marked increase in serum levels of GPT and GOT (two established biomarkers for liver damage), which pointed to liver injury. These results aligned with prior research demonstrating similar liver disturbances in tilapia fed on a HFD [29,39]. It has been reported that liver damage induced by HFD in different freshwater fish is related to both the species and dietary fat levels. In *Ctenopharyngodon idellus*, a diet with 8% fat significantly raised serum GPT and GOT levels after eight weeks [40]. Similarly, in the hybrid yellow catfish (*Tachysurus fulvidraco♀ × Pseudobagrus vachellii♂*), a diet with 15% fat led to a significant increase in both GPT and GOT activities after eight weeks [41]. On the other hand, *M. amblycephala* showed no changes in serum GPT and GOT levels with a 10% fat diet, yet experienced a significant increase in the two enzymes when the fat content was raised to 15%, after six weeks [42]. In *O. niloticus*, a diet containing 18.5% lipid increased GOT without altering GPT after 60 days [43].

TC and TG are critical components of fish blood lipids and play a vital role as diagnostic markers in pathology. They are indicative of both the lipid metabolism in the liver and the overall health status of the fish. Elevation in blood TC and TG after consuming a HFD may suggest a disruption in lipid metabolism [44]. In the current study, serum TC and TG levels also showed marked increases, indicating that HFD feeding led to lipid overload and disrupted lipid metabolism. These findings are consistent with previous research across different species, for instance, in *M. amblycephala*, where HFD feeding significantly elevated plasma TG levels [45], and in *Cyprinus carpio*, where serum TG and TC levels increased significantly after 8 weeks of HFD feeding [46].

### 4.2. The Influence of HFD on Energy Metabolism

Aquatic animals exhibit physiological adaptation in response to alteration in their environmental conditions, necessitating the consumption of additional energy. The production of cellular ATP, essential for this process, relies on oxidative phosphorylation in the mitochondrial electron transport chain. HFD feeding has been found to reduce the activity of oxidative phosphorylation complexes and induces nonalcoholic steatohepatitis in mice [47]. Previous studies have demonstrated significant reduction in the activity of oxidative phosphorylation complexes in patients with non-alcoholic steatohepatitis [48] and ob/ob mice [49]. However, some studies have observed that a HFD increased the content of oxidative phosphorylation proteins in mouse liver [50]. In our study, GSEA demonstrated an upregulated trend in the oxidative phosphorylation pathway, potentially enhancing ATP production. Nevertheless, research on the contributions of altered oxidative phosphorylation in liver metabolic disorders presents inconsistent results. For example, mitochondria facilitated hepatocyte proliferation by supplying ATP through oxidative phosphorylation post-hepatectomy [51]. Enhancing mitochondrial function via drugs has proven effective in improving non-alcoholic fatty liver disease [52]. Conversely, a reduction in mitochondrial oxidative phosphorylation may protect against diet-induced steatosis and slow the progression of non-alcoholic steatohepatitis [53]. Therefore, it remains unclear whether the upregulation of the oxidative phosphorylation pathway in this study constitutes a beneficial adaptation or exacerbates hepatic steatosis, highlighting the need for further investigation in fish.

In fish, glucose is central to metabolic processes, primarily via its conversion through glycolysis—a pathway essential for ATP production and providing precursors for various biosynthetic processes [54]. Glucose metabolic imbalance is commonly observed in animals fed a HFD [55]. Specifically, in mice, HFD impaired glucose homeostasis and altered insulin sensitivity in the liver [56]. In glucose metabolism, glucokinase (GCK) catalyzes a critical reaction by converting glucose into glucose-6-phosphate [57]. Previous studies have shown that a HFD can have varying effects on GCK activity in different species. While GCK activity was found to decrease in the liver of rainbow trout (*Oncorhynchus mykiss*) fed a HFD [58], contrasting results have been reported with an upregulation of *gck* expression observed in *M. amblycephala* on a similar diet [45]. In the present study, we observed that a HFD led to downregulated *gck* expression in the liver, suggesting that HFD feeding may inhibit glycolysis.

The transport of glucose across plasma membranes is regulated by glucose transporters (GLUTs), a family of transmembrane glycoproteins [59]. GLUT1, highly expressed in several tissues, facilitates the entry of glucose in metabolic processes crucial for maintaining normal insulin secretion [60,61,62,63]. Increased lipid availability in obesity leads to altered GLUT expression. Jha et al. (2019) found that a HFD led to increased GLUT1 expression, which might be linked to an imbalance in glucose homeostasis and contributed to insulin resistance in the livers of mice [64]. In *Haliotis discus hannai*, HFD feeding upregulated the *glut1* mRNA level in hepatopancreas [65]. Similarly, the present study found that *glut1* expression was upregulated in the liver after HFD feeding, potentially as an adaptive mechanism to maintain glucose homeostasis [66]. However, it is also suggested that the overexpression of GLUT1 can significantly contribute to glycoxicity, leading to the production of reactive oxygen species (ROS) in the liver [62].

Glucosamine-phosphate N-acetyltransferase 1 (GNPNAT1) is an essential enzyme in glucose metabolism, involved in the hexosamine pathway that produces UDP-N-acetylglucosamine and glutamate from glucose [67]. GNPNAT1 contributes to insulin secretion and influences cell cycle progression. Deficiency or inactivation of GNPNAT1 results in delayed cell cycles, ultimately leading to cell death [67]. The study demonstrated that *gnpnat1* expression was upregulated after HFD feeding, potentially promoting glucose metabolism [68]. PCK1, a key enzyme in the liver, facilitates the transformation of oxaloacetate (OAA) and GTP into phosphoenolpyruvate, a critical reaction that marks the first rate-limiting step of gluconeogenesis [69]. PCK1 is crucial in liver metabolic disease progression, as mice with targeted liver deficiency of PCK1 display hepatic lipid disorders and liver injury [70]. The study observed an upregulation of *pck1* in the HFD group, which suggested that this upregulation may play a role in maintaining glucose homeostasis in the liver under adverse conditions.

### 4.3. The Influence of HFD on Lipid Metabolism

It is well-known that HFD feeding causes disruptions in lipid metabolism in the liver of fish. HFD feeding inhibits fatty acid β-oxidation, enhances de novo synthesis of fatty acids, and promotes TG synthesis [28,71,72,73]. In the present study, GO enrichment analysis revealed that lipid metabolic processes were significantly altered following HFD feeding. Notably, KEGG analysis demonstrated marked changes in steroid biosynthesis, which exhibited a downward trend after HFD treatment. Steroid hormones are pivotal in regulating metabolism and stress responses. They also contribute to the development of diet-induced obesity by affecting the metabolism of fasting triglycerides [74], and increase circulating fatty acid levels by boosting dietary fat intake and enhancing the hydrolysis of triglycerides via lipoprotein lipase activity [75]. Furthermore, steroids have been shown to mediate liver steatosis in mice [76]. Alteration in the level of steroid hormones can lead to various degrees of hepatocellular damage [77]. In the work, downregulation of steroid biosynthesis may be associated with excessive lipid accumulation in liver triggered by HFD, which may adversely impact the normal liver metabolic functions.

### 4.4. The Influence of HFD on Protein Synthase

Proteins are essential components of all cells, and their synthesis is a complex, energy-consuming process that includes several stages: transcription, translation, protein folding, trafficking, and degradation [78]. The ribosome, a complex macromolecular machine, is crucial in protein synthesis. Transport of proteins across the endoplasmic reticulum (ER) membrane constitutes a critical initial step in the biosynthesis of many eukaryotic proteins. As a vital cytoplasmic organelle, the ER not only facilitates protein transport but also ensures the proper folding of proteins that are either transiting through or resident within it. HFD has been shown to reduce muscle protein synthesis [79]. Additionally, hepatic rRNA transcription—a key process in ribosome biogenesis—was found to be repressed by HFD feeding, which is essential for energy storage [80]. However, in our study, GSEA results indicated that the pathways for ribosome synthesis, protein transport, and protein processing in the endoplasmic reticulum exhibited upregulated trends in fish treated with a HFD. The specific reasons behind this phenomenon warrant further investigation. This upregulation of protein synthesis could potentially lead to increased energy consumption.

## 5. Conclusions

In summary, our study demonstrated that a HFD led to alterations in energy, lipid, and protein metabolic functions in the liver of tilapia. HFD feeding resulted in increased activities of GPT and GOT, and elevated levels of TG and TC, indicating liver injury and lipid deposition. Additionally, the HFD altered hepatic glucose homeostasis, as evidenced by the downregulation of *gck* and the upregulation of *glut1*, *gnpat1*, and *pck1*. Transcriptome analysis showed that in the HFD-fed group, 538 genes were markedly downregulated, while 460 genes were clearly upregulated. These DEGs predominantly participated in lipid metabolic processes and monocarboxylic acid metabolic processes. Furthermore, HFD feeding caused significant alterations in pathways related to steroid biosynthesis, porphyrin metabolism, terpenoid backbone biosynthesis, and retinol metabolism. Moreover, GSEA indicated a positive enrichment in pathways including oxidative phosphorylation, protein export, protein processing in the endoplasmic reticulum, and ribosome biogenesis in the HFD group. These findings enhance our understanding of the mechanisms behind HFD-induced hepatic dysfunction in fish.

## Figures and Tables

**Table 1:** The specific primer information in the study.

Gene Name	Primer Sequence (5′-3′)	GenBank Number/References	Product Length (bp)	Tm (°C)	Amplification Efficiency (%)
Glucosamine-phosphate N-acetyltransferase 1 (*gnpnat1*)	F: GAAGTCGTCGTCAGCGATGT	XM_003437497.4	118	60.45	97.3
	R: TGGGTGCACATTCAAGAGTGA			59.86	
Glucose transporter 1 (*glut1*)	F: AGTCTGCAATCAACTGGCCTC	FJ914657.1	249	60.61	98.6
	R: CCCATCTGGTGGAGTGACATAG			59.9	
ATP binding cassette subfamily F member 2 (*abcf2*)	F: GACCCAATGGAGCTGGGAAA	XM_005448995.3	131	59.96	97.9
	R: CAGTTGCTCAGTCAGGTGCT			60.25	
Glucokinase (*gck*)	F: CTGTGACATTGTGCGTCTGG	XM_003451020.5	101	59.49	99.3
	R: GTCTCTCCCGCATCAGGTTG			60.46	
Phosphoenolpyruvate carboxykinase 1 (*pck1*)	F: CGCATTCTGGACTGGATGTTC	XM_003448375.4	181	59.33	99.7
	R: TCCTGATCTCATCCACCTCCC			60.41	
Glutamine synthase a (*gs*)	F: AGCTACCACATTCGTGCCTAC	NM_001279668.1	139	60.13	101.2
	R: TACGAGGAATGCGAATGCTGG			60.81	
NADH-cytochrome b5 reductase 2 (*cbr2*)	F: ATCGCTGGTGGAACAGGTATC	XM_003439423.3	200	59.86	98.8
	R: TGTGGAGGTTTGTCCAGTGT			59.08	
Cytochrome c oxidase subunit I (*cox1*)	F: GGCCGGGGTGTCATCTATTT	NC_013663	154	59.82	101.6
	R: GGCAAGAACGGGTAGGGATAG			59.93	
Ubiquitin-conjugating enzyme (*ubce*)	F: CTCTCAAATCAATGCCACTTCC	[38]	130	57.63	103.8
	R: CCCTGGTGGAGGTTCCTTGT			61.43	

**Table 2:** Changes in serum parameters in tilapia after HFD feeding.

Parameters	Ctr Group	HFD Group	*p*-Value
GPT (U/L)	13.17 ± 1.26	25.66 ± 2.07	<0.001
GOT (U/L)	6.92 ± 1.35	15.02 ± 2.67	0.019
TG (mmol/L)	1.89 ± 0.08	3.60 ± 0.41	0.001
TC (mmol/L)	6.49 ± 0.55	19.72 ± 2.18	<0.001

The values are expressed as means ± SEM (*n* = 9).

**Table 3:** Statistical table of RNA-sequencing data.

Samples	Raw Reads	Clean Reads	Q_20_ (%)	Q_30_ (%)	GC (%)	Total Mapping Ratio (%)
Ctr-1	41,161,756	40,460,582 (98.3%)	98.17	94.28	50.01	89.45
Ctr-2	51,320,144	50,572,504 (98.54%)	98.30	94.58	50.04	87.50
Ctr-3	43,276,462	42,573,850 (98.38%)	98.23	94.41	50.39	88.17
HFD-1	32,736,674	32,146,546 (98.2%)	98.01	93.85	50.31	89.30
HFD-2	41,827,178	41,095,560 (98.25%)	98.21	94.36	50.09	88.43
HFD-3	42,561,088	41,819,868 (98.26%)	98.06	94.02	50.69	87.67

Note: Q_20_ and Q_30_, the base quality score was no less than 20 and 30, respectively, in clean reads. GC, GC content in clean reads.

## Data Availability

All data are contained within the main manuscript. The raw sequencing data have been uploaded to the NCBI Open database (PRJNA1170218).
